# Optoelectronic parametric oscillator

**DOI:** 10.1038/s41377-020-0337-5

**Published:** 2020-06-15

**Authors:** Tengfei Hao, Qizhuang Cen, Shanhong Guan, Wei Li, Yitang Dai, Ninghua Zhu, Ming Li

**Affiliations:** 1 State Key Laboratory on Integrated Optoelectronics, Institute of Semiconductors, Chinese Academy of Sciences, Beijing, 100083 China; 2 School of Electronic, Electrical and Communication Engineering, University of Chinese Academy of Sciences, Beijing, 100049 China; 3 State Key Laboratory of Information Photonics and Optical Communications, Beijing University of Posts and Telecommunications, Beijing, 100876 China

**Keywords:** Microwave photonics, Optoelectronic devices and components

## Abstract

Oscillators are one of the key elements in various applications as a signal source to generate periodic oscillations. Among them, an optical parametric oscillator (OPO) is a driven harmonic oscillator based on parametric frequency conversion in an optical cavity, which has been widely investigated as a coherent light source with an extremely wide wavelength tuning range. However, steady oscillation in an OPO is confined by the cavity delay, which leads to difficulty in frequency tuning, and the frequency tuning is discrete with the minimum tuning step determined by the cavity delay. Here, we propose and demonstrate a counterpart of an OPO in the optoelectronic domain, i.e., an optoelectronic parametric oscillator (OEPO) based on parametric frequency conversion in an optoelectronic cavity to generate microwave signals. Owing to the unique energy-transition process in the optoelectronic cavity, the phase evolution in the OEPO is not linear, leading to steady single-mode oscillation or multimode oscillation that is not bounded by the cavity delay. Furthermore, the multimode oscillation in the OEPO is stable and easy to realize owing to the phase control of the parametric frequency-conversion process in the optoelectronic cavity, while stable multimode oscillation is difficult to achieve in conventional oscillators such as an optoelectronic oscillator (OEO) or an OPO due to the mode-hopping and mode-competition effect. The proposed OEPO has great potential in applications such as microwave signal generation, oscillator-based computation, and radio-frequency phase-stable transfer.

## A new oscillator featuring phase-controlled oscillation

Parametric oscillators are driven harmonic oscillators that widely used in various areas of applications. In the past, parametric oscillators have been designed in the pure optical domain or the electrical domain, which are both delay-controlled oscillators with a steady oscillation confined by the cavity delay. Ming Li from the Chinese Academy of Sciences in Beijing and his colleagues have now developed a brand-new parametric oscillator in the microwave photonics domain, i.e., a hybrid optical-electrical oscillator. Owing to the unique parametric process in the optoelectronics cavity, the oscillation in the optoelectronic parametric oscillator is a phase-controlled operation, leading to a steady oscillation that is not bounded by the cavity delay. Continuously tuneable single frequency oscillation and stable multimode oscillation are produced by the new optoelectronic parametric oscillator, which are hard or even impossible to achieve in traditional delay-controlled oscillators.

## Introduction

Oscillators are widely used in all aspects of modern society, from watches and mobile phones to high-energy physics experiments, as well as in gravitational wave detection^1–5^. Parametric oscillators are an important type of oscillator based on a nonlinear process. In particular, optical parametric oscillators (OPOs) greatly extend the operating frequency of lasers by utilizing second-order or third-order nonlinearity, while the operating frequency range of ordinary lasers is limited to the stimulated atomic energy level^6–9^. However, an OPO, especially the doubly resonant OPO (DRO)^10^, is difficult to operate because not only the phase-matching condition but also the mode condition for the signal and idler should be satisfied. Steady oscillation in such an OPO is a delay-controlled operation, which is confined by the cavity delay since the signal must repeat itself after each round trip if any timing jitter is ignored. The delay-controlled operation leads to difficulty in frequency tuning, and the frequency tuning is discrete with the minimum tuning step determined by the cavity delay^11^. On the other hand, one of the attractive features of an OPO is its continuous tunability, which is generally implemented by changing the temperature, orientation or poling period of the crystal to influence the phase-matching conditions^12–14^. These approaches essentially change the round-trip time of the lightwave. The cavity modes are still determined by the cavity delay, similar to other delay-line oscillators. A parametric oscillator can also be designed in the radio-frequency (RF) domain by utilizing a nonlinear electronic device^15,16^, e.g., a varactor diode. In practice, the parametric process in the RF domain is used to amplify weak signals with ultralow noise, which is very important in areas such as long-range radar, radio telescopes, and satellite ground stations. Essentially, the conventional parametric process in the RF domain has no difference from that in the optical domain. On the other hand, an optoelectronic oscillator (OEO) is another type of delay-line oscillator that is implemented in an optoelectronic cavity^17–20^. It has a hybrid positive feedback loop formed by an optical path and an electrical path to create microwave signals with ultralow phase noise owing to the use of a high quality-factor (Q factor) optical energy-storage element, such as a long optical fiber delay line. The generation of ultrastable single-mode microwave signals and multimode microwave oscillations, such as broadband chaotic signals, has been widely demonstrated using OEOs. Nevertheless, steady oscillation in an OEO is also a delay-controlled operation, which is also confined by the cavity delay as in an OPO and an electrical parametric oscillator.

Here, we propose an optoelectronic parametric oscillator (OEPO) based on the second-order nonlinearity in an optoelectronic cavity. A pair of oscillation modes is converted into each other in the nonlinear medium by a local oscillator (LO) in the proposed OEPO. The sum phase of each mode pair is locked by the LO, which ensures stable multimode oscillation that is difficult to realize in conventional oscillators such as an OEO or an OPO due to the mode-hopping and mode-competition effect. Moreover, owing to the unique energy-transition process in the optoelectronic cavity, oscillation in the OEPO is a phase-controlled operation, whose frequency can be independent of the cavity delay. Continuous frequency tuning is achieved without the need for modification of the cavity delay. These unique and remarkable features of the proposed OEPO make it a strong competitor in applications such as microwave signal generation, oscillator-based computation, and radio-frequency phase-stable transfer.

## Results

**Fig. 1:**
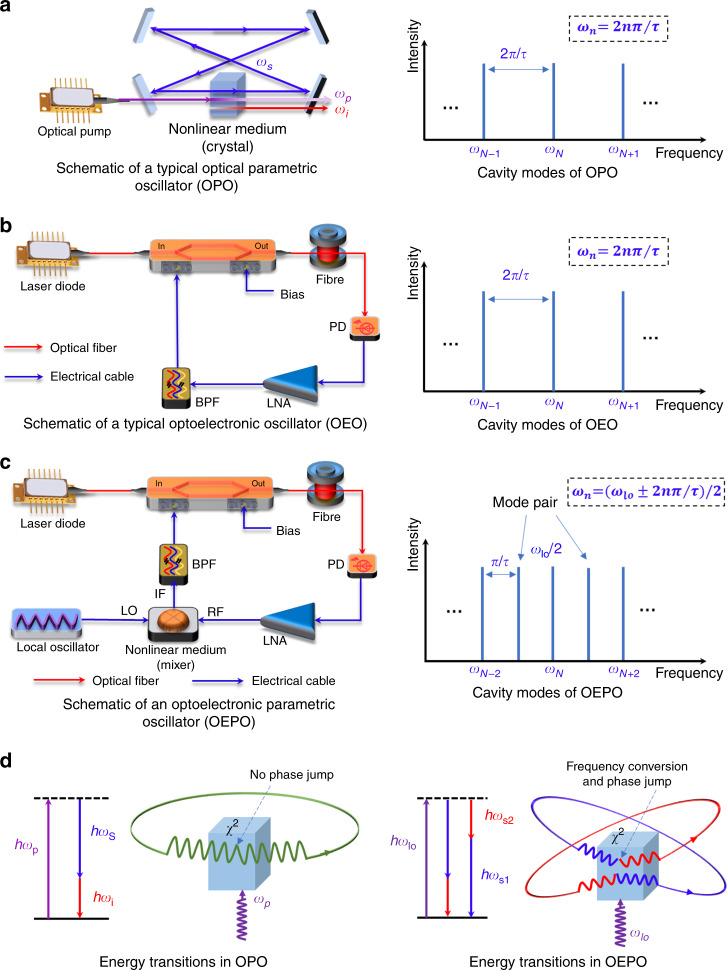
Comparison between an OPO, an OEO and the proposed OEPO. **a** Schematic and cavity modes of a typical OPO. The cavity modes are discrete, and the minimum mode spacing is 2*π*/τ, where τ is the cavity delay. **b** Schematic and cavity modes of a typical OEO. The cavity modes are similar to those of an OPO since they are both delay-controlled oscillators. **c** Schematic and cavity modes of the proposed OEPO. *ω*_*lo*_ is the frequency of the LO. Due to the phase jump in the parametric frequency-conversion process, the cavity modes can be continuously tuned by tuning the LO. The minimum mode spacing is *π*/τ. **d** Energy transitions in a typical OPO and in the proposed OEPO. Oscillation in the OPO is based on optical parametric amplification. The energy flows from the pump to the signal and idler through an optical nonlinear medium. There is no phase jump for the oscillating signals in the optical nonlinear medium. In the proposed OEPO, oscillation is based on electrical parametric frequency conversion. A pair of oscillations are converted into each other in the electrical nonlinear medium by the local oscillator (LO). There is a phase jump for the oscillating signals in the nonlinear medium, which leads to the unique mode properties of the proposed OEPO. PD photodetector, LNA low noise amplifier, BPF bandpass filter

A comparison between an OPO, an OEO and the proposed OEPO is shown in Fig. 1. In the OPO, energy flows from the pump *ω*_*p*_ to the signal *ω*_*s*_ and idler *ω*_*i*_ through an optical nonlinear medium, such as an optical nonlinear crystal. The signal and idler are amplified by the optical gain arising from the parametric amplification in the optical nonlinear medium, which allows one or both of them to oscillate in the OPO. In addition to the optical nonlinear medium, an optical resonant cavity is also an essential component of the OPO. The optical resonant cavity serves to resonant at least one of the signal and idler wavelengths. If the resonant cavity is resonant at either the signal or idler wavelength, then it is a singly resonant OPO (SRO). When both the signal and idler are resonant, then the cavity is a DRO. Another configuration is a triply resonant OPO (TRO), in which the pump, signal and idler waves are resonant simultaneously. One of the most attractive features of an OPO is that it is possible to access wavelengths that are difficult or even impossible to obtain with lasers, such as in the mid-infrared spectral region. Moreover, wide wavelength tunability is also possible by changing the pump wavelength or the phase-matching properties, which is highly desired in practical applications such as laser spectroscopy. However, the wavelength tuning in an OPO is generally complicated because the operation wavelengths are determined not only by the phase-matching condition but also primarily by the requirement for resonance of the oscillating signal in the OPO cavity. In steady oscillation, the signal must repeat itself after each round trip if any timing jitter is ignored, so only specific frequency components whose frequencies are *n/τ* can survive, where *n* is an integer, and *τ* is the cavity delay. Frequency tuning is discrete, and the minimum tuning step is the cavity free spectral range *FSR* = 1/*τ*. The cavity modes of an OEO are also discrete, and the minimum mode spacing is 2*π*/τ, similar to that of an OPO since they are both delay-controlled oscillators. In the physical configuration, the OEO and the proposed OEPO are both implemented in an optoelectronic cavity. The major difference is that a parametric frequency-conversion process is introduced into the OEPO cavity, which leads to the unique properties and advantages of the proposed OEPO. In addition, the oscillating signal in the OEO is established directly from noise, and there are no energy transitions from the pump signal to the oscillating signal, as in the proposed OEPO and OPO.

In the proposed OEPO, we use an electrical frequency mixer as the second-order nonlinearity device to realize a parametric frequency-conversion process in the optoelectronic cavity. The electrical frequency mixer is a nonlinear electrical device that produces new frequencies. Generally, two input signals $E_{lo}{\mathrm{cos}}\left( {\omega _{lo}t + \varphi } \right)$ and $E_{sig}{\mathrm{cos}}\left( {\omega _{sig}t} \right)$ are applied to a mixer, and the output signal can be expressed as $v_{out} \propto [E_{lo}{\mathrm{cos}}\left( {\omega _{lo}t + \varphi } \right) + E_{sig}{\mathrm{cos}}\left( {\omega _{sig}t} \right)]^2 \propto E_{lo}E_{sig}\cos \left[ {\left( {\omega _{lo} - \omega _{sig}} \right)t + \varphi } \right]$. Clearly, the mixer creates a new signal at the difference of the original frequencies. A pair of oscillations will be converted into each other in the electrical nonlinear medium by the LO in the proposed OEPO. Different from the conventional parametric oscillator where the pump provides the gain, the signal gain in our scheme is provided by an electrical amplifier, while the LO only frequency converts the input signal to another frequency component. This frequency conversion is unconditional, while the frequency conversion in an OPO requires the phase-matching condition. As shown in Fig. 1d, there is a phase jump for the oscillating signals in the nonlinear medium, which leads to unique mode properties of the proposed OEPO. Such a phase jump also locks the sum phase of each mode pair, so stable multimode oscillation can be expected in the OEPO cavity. This is a unique and remarkable feature of the proposed OEPO since stable multimode oscillation is difficult to achieve in a conventional OEO or OPO due to the mode-hopping and mode-competition effect. If the frequency of the signal generated in the parametric frequency-conversion process is not the same as the input frequency, then this parametric process is nondegenerate. In this case, steady oscillation must occur in the form of a mode pair, whose sum frequency equals the LO frequency. In the parametric frequency-conversion process, the LO converts these two modes into each other, so the oscillating signal can repeat itself after two round trips, as shown in the right part of Fig. 1d. The oscillation frequency of the OEPO can also be tuned easily, for example, by tuning the frequencies of the mode pairs by changing the frequency of the LO or by using different bandpass filters (BPFs) to select the desired frequency components from all cavity modes.

Mathematically, the oscillating mode pair in the optoelectronic cavity can be expressed as $s\left( {z,t} \right) = e^{ - i(\omega _{s1}t - k_{s1}z + \varphi _{s1})} + e^{ - i(\omega _{s2}t - k_{s2}z + \varphi _{s2})} + c.c.$, where *z* is the spatial position along the cavity (here, *z* = 0 is considered as the position where frequency conversion is implemented), *ω*_*s*1_ and *ω*_*s*2_ are the angular frequencies, *φ*_*s*1_ and *φ*_*s*2_ are the initial phases, and *k*_*s*1_ and *k*_*s*2_ are the wave vectors of the mode pair. Assuming that the pump from the LO is $p\left( {z = 0,t} \right) = p\left( {z = L,t} \right) = e^{ - i\omega _{lo}t} + c.c.$, according to the mode condition, the oscillating microwave *s*(*z,t*) should satisfy

1 $$\left\{ {\begin{array}{*{20}{c}} {s\left( {z = 0,t} \right) = \alpha _1p\left( {z = L,t} \right)s^ \ast \left( {z = L,t} \right) + c.c.} \\ {s\left( {z = L,t} \right) = \alpha _2s\left( {z = 0,t + \tau } \right) + n_a(t) + c.c.} \end{array}} \right.$$

where *α*_1_ is the frequency-conversion loss, *α*_2_ includes the loss from the electrical-to-optical (E/O) and optical-to-electrical (O/E) conversions, from the degeneracy when the signal propagates along the cavity, and from the gain provided by the low noise amplifier (LNA), and *n*_*a*_(*t*) is the additive noise including the thermal noise, shot noise and relative intensity noise (RIN) of the laser. Here, the noise in the frequency conversion is ignored. The total loop gain *G*_loop_ = *α*_1_*α*_1_ is near unity when the oscillation is in the steady state. Assuming that the additive noise in the optoelectronic cavity is negligible, according to Eq. (1), we obtain

2 $$\left\{ {\begin{array}{*{20}{c}} { - \omega _{s1}\tau - \varphi _{s1} + 2N_1\pi = \varphi _{s2}} \\ { - \omega _{s2}\tau - \varphi _{s2} + 2N_2\pi = \varphi _{s1}} \end{array}} \right.$$

where *N*_*1*_ and *N*_*2*_ are integers. Combined with the frequency relationship *ω*_*s*1_ + *ω*_*s*2_ = *ω*_*l*o_, we finally obtain

3 $$\left\{ {\begin{array}{*{20}{c}} {\omega _{s1},\omega _{s2} = \frac{{\omega _{lo} \pm 2N\pi \times FSR}}{2}} \\ {\varphi _{s1} + \varphi _{s2} = \frac{{ - \omega _{lo}\tau + 2M\pi }}{2}} \end{array}} \right.$$

where *N=N*_1_ − *N*_2_ and *M=N*_1_ + *N*_2_. We can see that the mode cluster is determined not only by the cavity delay but also by the LO. Modification of either of them would change the mode cluster. Moreover, the oscillating modes must appear symmetrically about half of the LO frequency. The frequency spacing between two cavity modes can be less than the *FSR*, that is, half of the *FSR*.

In the case of degenerate oscillation, the frequency-converted signal has the same frequency as the input signal, i.e., *ω*_*s*1_ = *ω*_*s*2_. According to Eq. (2), the oscillation frequency and its initial phase can be expressed as

4 $$\left\{ {\begin{array}{*{20}{c}} {\omega _s = \frac{{\omega _{lo}}}{2}} \\ {\varphi _s = - \frac{{\omega _{s}\tau }}{2} + K\pi } \end{array}} \right.$$

We can see that the oscillator outputs a single-frequency microwave whose frequency is fixed at *ω*_*lo*_/2. In this instance, the oscillation frequency can be completely independent of the cavity delay. Furthermore, the initial phase of the oscillating signal is locked to *φ*_*s*_ = −*ω*_*s*_τ/2 + *Kπ*, where *K* is an integer.

Why can such oscillation be independent of the cavity delay? In the conventional resonant cavity, the spatial boundary condition confines the cavity modes to integer multiples of the fundamental mode. In our proposed resonator, the parametric frequency conversion provides a phase jump for the oscillating modes. In the degenerate case, this phase jump is a result of the phase-conjugate operation that inverts the phase of the input signal to the opposite phase at the parametric frequency-conversion output. The phase evolution in the proposed OEPO is not linear owing to this phase jump, while the phase evolution in the conventional oscillator is linear or quasi-linear. A more detailed comparison of the phase evolution between the proposed OEPO and conventional oscillator can be found in Supplementary Note 1. The phase jump is 2*φ*_*s*_ = −*ω*_*s*_τ + 2*Kπ* since the initial phase is locked to *φ*_*s*_ = −*ω*_*s*_τ/2 + *Kπ*. One consequence of the phase jump is that continuous frequency tuning can be achieved, while modification of the cavity delay is not required. We can call such oscillation phase-controlled oscillation, while the conventional oscillation is delay-controlled oscillation. The phase-conjugate operation in the parametric process forces the signal to oscillate at half of the LO frequency regardless of the cavity delay. Different from the conventional oscillator, where the signal has a random phase because oscillation starts from noise, the degenerate oscillation in the OEPO has a specific initial phase relative to the LO. In the nondegenerate case, the parametric frequency conversion also leads to a phase jump of −*ω*_*lo*_τ/2 + *Mπ* for all the frequency components. As a result, the mode cluster is not restricted by the cavity delay. In fact, the cavity delay and the LO together determine the mode cluster. In contrast to the spatial boundary condition in the conventional resonator cavity, the oscillation has a phase condition, that is, the sum phase of the signals before and after the frequency conversion equals the phase of the LO.

**Fig. 2:**
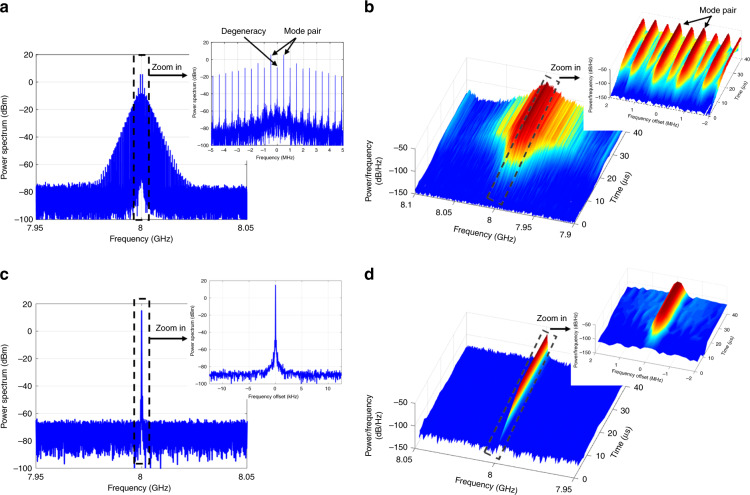
Power spectra and oscillation processes of the OEPO outputs in multimode and single-mode operation. **a** Power spectrum in multimode oscillation, which contains degenerate and nondegenerate oscillations. Inset graphic: details of the power spectrum in multimode oscillation. The nondegenerate oscillation is symmetric about the degenerate one, and the minimum mode space is 1/2 the cavity *FSR*. **b** Oscillation process in multimode oscillation. The two frequency components in each mode pair grow at the same rate and become stable simultaneously. **c** Power spectrum in single-mode oscillation, which is also degenerate oscillation. Inset graphic: details of the power spectrum in single-mode oscillation with a resolution bandwidth (RBW) of 100 Hz. The OEPO has a narrow linewidth that is smaller than the RBW in the inset graphic. **d** Oscillation process in degenerate oscillation

The power spectrum and oscillation process of multimode oscillation in the OEPO are shown in Fig. 2a, b. The cavity *FSR* in our experiment is 1 MHz. As our theory predicts, the oscillating modes of the multimode oscillation are symmetrical about half of the external frequency, and the minimum mode spacing is half of the cavity *FSR*. In addition, the two frequency components of each mode pair have the same power. From the real-time frequency distribution in Fig. 2b, we can further conclude that the signal oscillation starts from noise, and the frequency components in each mode pair emerge and becomes stable simultaneously. It should be mentioned that nondegenerate oscillation in OEPO is easy to realize and occurs stably without mode hopping in our experiment, while stable multimode oscillation is difficult in a conventional OEO or OPO due to the mode-hopping and mode-competition effect. In an OEO, multimode oscillation is achieved by using a loop filter with a large bandwidth to select a number of cavity modes. This multimode oscillation is not stable and even chaotic under large feedback gain due to the mode-hopping and mode-competition effect^21,22^. In an OPO, it is often assumed that the resonant cavity allows only one or two modes, depending on whether degenerate or nondegenerate operation is considered. However, multimode oscillation will also occur at high pump powers, and its stability is poor due to the mode-competition effect^23,24^. Generally, stable multimode oscillation in an OEO or an OPO requires a mode-locking mechanism, e.g., Fourier domain mode locking^25,26^ or a saturable absorber^27^. A specific mode-locking mechanism locks the modes in a specific phase and amplitude relationship so that the oscillator outputs the designed waveform. In the OEPO, the phase control by the LO locks the sum phase of each mode pair, so stable multimode oscillation can be easily achieved. The proposed OEPO can therefore be applied in scenarios requiring stable, wideband, and complex microwave waveforms.

**Fig. 3:**
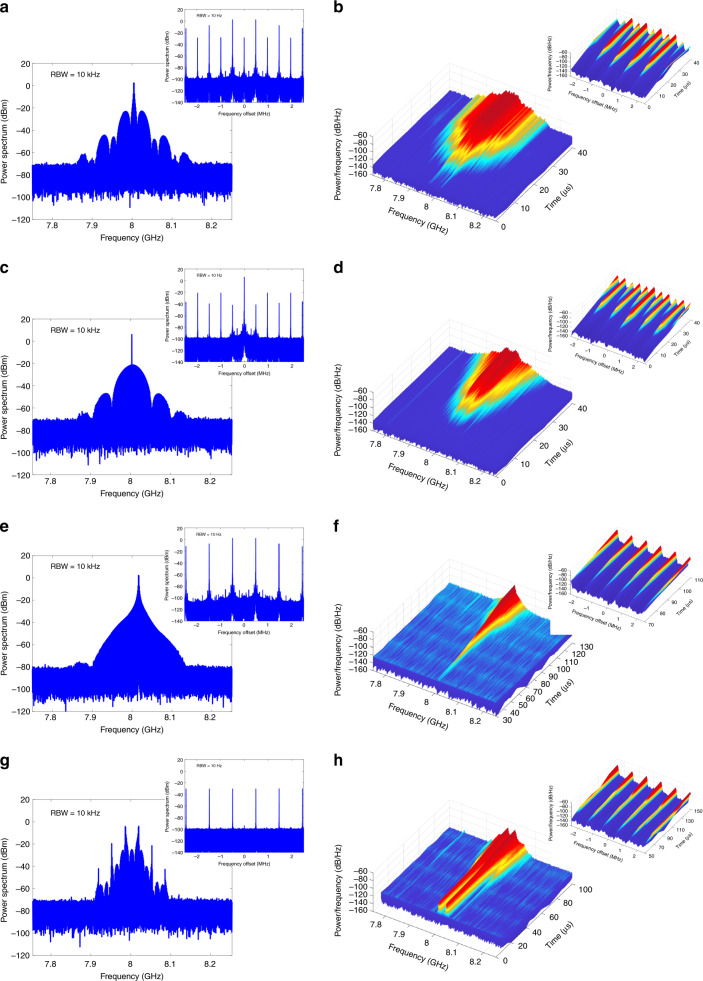
Power spectra and oscillation processes of the multimode OEPO with different spectral envelopes and frequency spacings of adjacent oscillation modes. The parameters, such as the cavity gain and pump frequency of the OEPO, are tuned to achieve these multimode oscillation states. **a**, **c**, **e**, **g** Power spectra. **b**, **d**, **f**, **h** Oscillation processes. RBW resolution bandwidth

At the same time, although the multimode oscillation is stable, it is still hard to predict the spectral envelope and frequency spacing of adjacent oscillation modes of the generated waveform because the oscillation starts from noise and the initial phases of the respective frequency components in a mode pair are not determined. In other words, the parametric process in the optoelectronic cavity only locks the sum phase of each mode pair, which ensures stable multimode oscillation when the parameters of the OEPO cavity are fixed. There are still many possible values for the initial phases of the respective frequency components in a mode pair because only their sum phase is determined. The initial phases are related to the parameters of the OEPO cavity, such as the cavity gain and pump frequency. Different multimode operation states can be achieved by tuning the parameters of the OEPO cavity. Several typical oscillation processes along with their power spectra are given in Fig. 3, when the cavity gain is changed or the pump frequency is tuned. We can see that in the cases of Fig. 3a, c, the frequency spacings of adjacent oscillation modes are both half of the cavity *FSR*, although they have different spectral envelopes. In the cases of Fig. 3e, g, the frequency spacings of adjacent oscillation modes equal the cavity *FSR*. In fact, the oscillating mode spacing can be larger than the cavity *FSR* as long as the initial phase and frequency of the oscillation modes satisfy Eq. (3). Nevertheless, as we can see from the above multimode power spectra and oscillation processes, the oscillation modes of the OEPO must oscillate in pairs and start at the same time with the same amplitude, which is consistent with our theory.

**Fig. 4:**
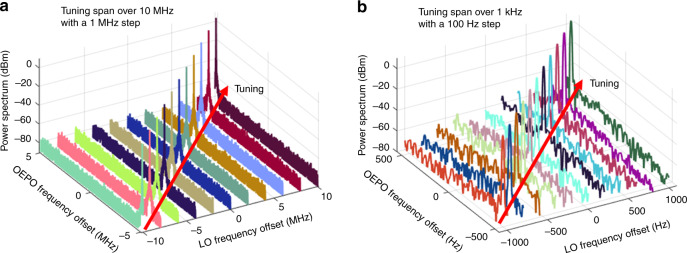
Cavity-delay-independent frequency tuning in the OEPO. The cavity length is approximately 200 m, resulting in a 1-MHz *FSR*. By tuning the LO, the frequency of the degenerate oscillation is also tuned. **a** Tuning step of 1 MHz. RBW: 1 kHz. **b** Tuning step of 100 Hz. RBW: 10 Hz

Figure 2c, d shows the power spectrum and oscillation process of the degenerate oscillation. The degenerate oscillation is single-mode oscillation with a narrow linewidth that is smaller than the resolution bandwidth (RBW) in the inset graphic shown in Fig. 2c. Demonstration of the delay-independent mode cluster is also implemented with the degenerate oscillation. In this instance, continuous frequency tuning is realized, as shown in Fig. 4. As the degenerate oscillation is half-harmonic generation, a $2\Delta f$ modification in the LO leads to a $\Delta f$ frequency tuning. No modification is applied to the cavity delay. It should be noted that the tuning step in our experiment is 100 Hz, which is much less than the 1-MHz cavity *FSR*. Moreover, it can be inferred from Fig. 4b that the linewidth of the single-mode oscillation is likely smaller than the smallest RBW of the electrical spectrum analyzer, which is 10 Hz. It should be noted that the function and structure of the OEPO are similar to those of an electrical frequency divider in this case. Frequency dividers based on electrical parametric oscillators have been reported previously^28,29^. The basic idea is to synchronize the oscillation frequency of the parametric oscillator to one of the subharmonics of the pump signal, which is similar to the degenerate oscillation in the proposed OEPO. However, the operating frequency range of these electrical parametric oscillators is limited by the nonlinear electronic device to several GHz. The operating frequency range of the proposed OEPO can be as large as tens of GHz, which is only limited by the bandwidth of the optoelectronic devices in the OEPO cavity.

## Discussion

Owing to the introduction of the parametric frequency-conversion process, the phase evolution in the OEPO cavity is not linear, leading to the unique and remarkable characteristics of the proposed OEPO compared with traditional oscillators such as the OEO and OPO. Stable and tuneable multimode oscillation is easy to realize without being bounded by the cavity delay due to the phase control of the parametric frequency-conversion process, while stable multimode oscillation is difficult to achieve in conventional oscillators, such as an OEO and an OPO, due to the mode-hopping and mode-competition effect. The OEPO can be applied in scenarios requiring stable, wideband, and complex microwave waveforms. By increasing the cavity length of the OEPO, the mode spacing of the optoelectronic cavity can be as small as several kHz, which would allow many densely distributed modes to oscillate in the cavity simultaneously. Oscillator-based computation can benefit from such a large mode number^30–33^. Furthermore, when the OEPO operates in degenerate mode, the parametric frequency conversion actually works as a phase-conjugate operation, similar to the phase-conjugate mirror in an OPO^34,35^. Based on this phase-conjugate operation, the phase error resulting from cavity turbulence can be autoaligned. Therefore, the OEPO can be used for phase-stable RF transfer (see details in Supplementary Information).

The noise characteristic is another important characteristic of an oscillator. In an optical parametric DRO, although the signal and the idler are mutually coherent with the pump and are phase anticorrelated with each other away from the degeneracy, they still suffer from cavity instability^36^. In the OEPO, we can expect that the coherence properties of a mode pair are similar to those in an optical parametric DRO since the sum phase of the mode pair is locked to the LO phase. In a self-excited oscillator, the Q factor of the resonator determines the purity of the output signal. This is why OEOs are so attractive for the generation of pure microwave signals^17–20^. In OEOs, the phase noise can be written as^37^

5 $$S_{\varphi 1}\left( f \right) = \frac{{S_{\varphi - link}\left( f \right)}}{{\left| {1 - H_d\left( f \right)H_f\left( f \right)} \right|^2}}$$

where $S_{\varphi - link}\left( f \right)$ is the open-loop residual phase noise associated with the residual phase noise of the electrical amplifier, the relative intensity noise (RIN) and frequency noise of the laser and the flicker noise of the photodetector. $H_d\left( f \right) = \exp \left( { - 2i\pi f\tau } \right) = \exp \left( { - iQ} \right)$ is related to the Q factor of the energy-storage medium, and *τ* is the delay induced by the energy-storage medium. *H*_*f*_ (*f*) is related to the loop filter. As can be seen from Eq. (5), by utilizing a low-loss fiber as the energy-storage medium, OEOs can obtain a high-Q factor and generate ultralow-noise microwave signals. The frequency components in the OEPO can also benefit from a high-Q-factor optoelectronic resonator cavity. In the case of degeneracy, since the oscillation is half-harmonic generation, the noise is only determined by the LO, and is independent of the cavity Q factor. In this case, assuming that the phase of the LO is $\psi _p = \omega _{lo}t + \Delta \psi _{lo}$, where $\Delta \psi _{lo}$ is the phase jitter of the LO, the phase of the degenerate oscillation can be expressed as $\psi _s = \omega _{lo}t/2 + \Delta \psi _{lo}/2 + \varphi _s$. Therefore, the frequency and phase jitter of the degenerate oscillation are half those of the LO. Considering the relationship between the phase noise and phase jitter $\Delta \psi = \sqrt {2\cdot 10^{A/10}}$^38^, where *A* is the integrated phase noise power, we can expect that the phase noise in the degeneracy is 20log_10_2 ≈ 6 dB lower than that of the LO. It is worth noting that phase noise reduction is quite common in the coherent frequency-division process since the phase jitter is also divided by *N* when the frequency is coherently divided by *N*. As a result, a phase noise reduction of 20log_10_*N* is achieved. In the nondegenerate oscillation of the proposed OEPO, as the mode pairs are frequency-converted by the LO, we can expect that their sum-frequency signal shares the same noise as the LO. The noise feature of the two frequency components in each mode pair is related to the LO noise feature, the cavity Q factor and the open-loop residual phase noise. The phase noise of the two frequency components can be written as

6 $$S_{\varphi 2}\left( f \right) = \frac{{S_{\varphi - link}\left( f \right)}}{{\left| {1 - H_d\left( f \right)H_f\left( f \right)} \right|^2}} \;+\; \frac{{S_{\varphi - lo}\left( f \right)}}{4}$$

where *S*_*φ–lo*_(*f*) is the phase noise of the LO. As can be seen, the phase noise of the two frequency components in each mode pair share the same noise feature. A better phase noise performance can be achieved by using a long fiber delay line to obtain a high-Q factor or by reducing the noise brought about by the active components such as the electronic amplifier.

**Fig. 5:**
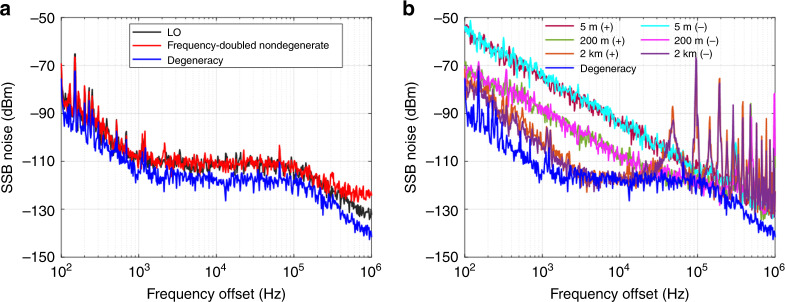
Noise feature of the OEPO. **a** Comparison of the single-sideband (SSB) noise between the LO, the degenerate oscillation, and the frequency-doubled nondegenerate oscillation. The results indicate that oscillation in the OEPO is phase-locked to the LO. **b** Comparison of the SSB noise from different Q-factor oscillations. As the Q factor increases, the SSB noise decreases. However, the degeneracy sets the lower bound

The coherence and noise features of the OEPO are evaluated by measuring the single-sideband (SSB) phase noise of the generated signal and the LO, and the results are shown in Fig. 5. In the nondegenerate oscillation, we use a frequency doubler to extract the sum phase of a mode pair. Although the frequency-doubled signal contains many frequency components, the frequency component at the pump frequency is only contributed by the frequency components from the same mode pair. To evaluate the noise characteristics over a larger frequency range, the fiber in the optoelectronic cavity is shortened to 5 m so that the sum frequency of frequency components from different mode pairs falls outside the observation span. We can see that the SSB noise of the frequency-doubled 8-GHz microwave is almost the same as that of the LO. This consistency proves that the sum phase of each mode pair is locked to the pump phase. Particularly, in the degenerate oscillation, the SSB noise is ~6 dB lower than the pump noise. As we mentioned above, the noise feature of the nondegenerate oscillation relates to the LO noise feature, the cavity Q factor and the open-loop residual phase noise. The degeneracy sets the lowest level limit for each frequency component. Moreover, the two frequency components in each mode pair share the same noise feature. Similar to the conventional OEO, a larger cavity delay increases the cavity Q factor and ultimately results in better noise performance. The SSB noise is measured for different cavity delays, and the results are shown in Fig. 5b. We can see that a larger cavity delay decreases the SSB noise. In the 2-km OEPO, the degeneracy sets a bound on the lowest potential SSB noise level. The two frequency components in each mode pair share the same SSB noise. In fact, the noise in each frequency component contains two parts: the correlated component and the anticorrelated component. The correlated noise comes from the LO, and is distributed equally among the two frequency components in a mode pair. As a result, the correlated noise has the same level as the degeneracy. The anticorrelated noise comes from the optoelectronic cavity, and its magnitude depends on the cavity Q factor and the active devices in the loop such as the electrical amplifier. In the frequency doubling, the anticorrelated noise of the two frequency components in each mode pair undergoes fully destructive interference, and the correlated noise undergoes constructive interference. As a result, the frequency-doubled signal has the same noise as the LO, while the degeneracy sets the lower bound for each frequency component.

In conclusion, we have demonstrated a new OEPO based on parametric frequency conversion in an optoelectronic cavity. In this new OEPO, the mode condition is no longer dependent on the traditional boundary condition. The cavity modes in the OEPO are discussed. We found that the cavity modes are dependent on the cavity delay and the phase of the external pump. In particular, degenerate oscillation could be independent of the cavity delay. In the nondegenerate oscillation, modes must oscillate in pairs with the same amplitude symmetrically about half of the external frequency. The sum phase of each mode pair is also locked by the parametric frequency-conversion process, which ensures stable multimode oscillation that is difficult to achieve in conventional oscillators. The noise characteristic of the OEPO is also discussed. We found that although external microwaves limit the noise performance of the OEPO, a long fiber still contributes to lower noise oscillation. The unique and remarkable features of the proposed OEPO make it a strong competitor in applications such as microwave signal generation, oscillator-based computation, and radio-frequency phase-stable transfer.

## Materials and methods

The fiber length in our experiment is ~200 m, corresponding to an ordinary *FSR* of 1 MHz. The LO frequency is 16 GHz, with a power of approximately 10 dBm. An electrical BPF with an 80-MHz bandwidth centered at 8 GHz is used to block the sum frequency and other spurs generated from the electrical mixer. An electrical signal analyzer (ESA) and a real-time oscilloscope are used to measure the oscillator output. To realize degenerate oscillation while suppressing nondegenerate oscillation, an equivalent narrow BPF^39^ is embedded in the optoelectronic cavity (see details in Supplementary Information) to replace the 80-MHz BPF. The equivalent narrow BPF is tuneable and has a 3-dB bandwidth of 12 kHz.

## Supplementary information

Supplementary Information for Optoelectronic Parametric Oscillator
