# Supplementary material for: Optoelectronic parametric oscillator
Source: Light Sci Appl. 2020 Jun 15;9:102. doi: 10.1038/s41377-020-0337-5 (PMC7295800; doi:10.1038/s41377-020-0337-5)
Supplement: Supplementary file 1 — Supplementary Information for Optoelectronic Parametric Oscillator [file 41377_2020_337_MOESM1_ESM.docx]

Supplementary Information for

Optoelectronic Parametric Oscillator

Tengfei Hao, Qizhuang Cen, Shanhong Guan, Wei Li, Yitang Dai, Ninghua Zhu and Ming Li

**Optoelectronic Parametric Oscillator**

Authors:

Tengfei Hao^1,2†^, Qizhuang Cen^3†^, Shanhong Guan^3^, Wei Li^1,2^, Yitang Dai^3*^, Ninghua Zhu^1,2*^ and Ming Li^1,2*^

Affiliations:

^1^ State Key Laboratory on Integrated Optoelectronics, Institute of Semiconductors, Chinese Academy of Sciences, Beijing 100083, China

^2^ School of Electronic, Electrical and Communication Engineering, University of Chinese Academy of Sciences, Beijing 100049, China

^3^ State Key Laboratory of Information Photonics and Optical Communications, Beijing University of Posts and Telecommunications, Beijing 100876, China

E-mail:

†These authors contributed equally to this work.

*e-mail: ytdai@bupt.edu.cn; nhzhu@semi.ac.cn; [ml@semi.ac.cn](mailto:ml@semi.ac.cn)

## Mode conditions in OEPO

As the signal comes from noise and stable oscillation requires coherent interference, a stable oscillation means that the signal repeats its phase and amplitude at a certain period of time. In the conventional oscillator, the phase of the oscillating signal is considered to evolve linearly. As a result, the oscillating modes have an accumulated phase of *2Nπ* when it propagates along the cavity in a round-trip. The potential oscillating frequencies equal to *nFSR*, where n is an integer and *FSR* is the cavity free spectral range that defined by round-trip time delay *FSR=1/τ*. However, in the optoelectronic parametric oscillator (OEPO), the phase evolution of the oscillation can be nonlinear due to the parametric frequency conversion. As shown in Fig. S1, the accumulated phase during one round-trip consists of two parts, the linear part comes from propagation along the optoelectronic cavity and the nonlinear part comes from the parametric process. As a result, we can expect that the potential oscillating modes in the OEPO depend on two elements: the cavity delay and the pump frequency. As the same in optical parametric oscillator (OPO), the OEPO can also operate in degenerate state or non-degenerate state. In the degenerate state, the generated difference frequency signal shares the same frequency with the input signal. In this case, the oscillator can be a single-mode one that outputs a single-frequency microwave. In the non-degenerate state, the frequency of the generated difference frequency signal is different from the input one, so that the oscillator operates at multi-mode state. A non-degenerate oscillation consists at least two frequency components, whose sum frequency equals to the pump frequency. These two frequency components are called as a mode pair.


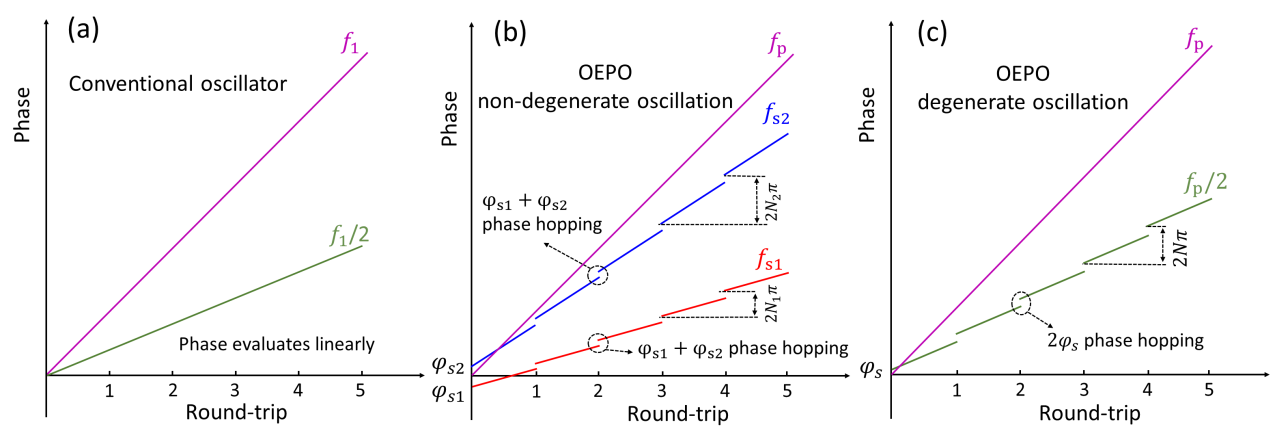


**Fig. S1**. A comparison of phase evolution between the proposed optoelectronic parametric oscillator (OEPO) and conventional oscillator. The phase evaluates linearly in conventional oscillator, while in the OEPO the phase can has a hopping. In the degenerate oscillation, the oscillating frequency is half of the pump. In the non-degenerate instance, oscillation occurs in mode pair, and the two frequency components from a mode pair experience the same phase hopping.

## Equivalent narrow bandpass filter


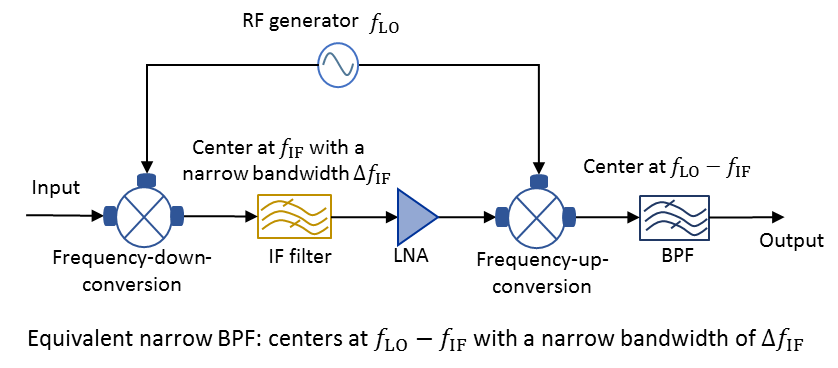


**Fig. S2**. The equivalent narrow bandpass filter (BPF). The center frequency of the equivalent BPF is determined by $f_{LO}-f_{IF}$, where $f_{LO}$ is the center frequency of the local oscillation (LO) and $f_{IF}$ is the center frequency of the intermediate frequency (IF) BPF. LNA, low noise amplifier.

The single-mode, degenerate oscillation is realized with the help of an equivalent narrow bandpass filter (BPF), as illustrated in Fig. S2. The signal is first frequency-down-converted to low frequency dominated by a local oscillation (LO), then filtered by a narrow intermediate frequency (IF) BPF in low frequency domain, and finally frequency-up-converted to recover the desired signal. A low noise amplifier (LNA) is used to compensate the loss of frequency conversions. The equivalent filter can achieve quite narrow bandwidth compared to that filter in high frequency domain. The center frequency of the equivalent BPF is determined by $f_{LO}-f_{IF}$, where $f_{LO}$ is the LO frequency and $f_{IF}$ is the center frequency of the IF-BPF. Therefore, the equivalent BPF can be a tunable one by adjusting the LO frequency. An IF filter with a bandwidth of 12 kHz is used in our experiment, so that the bandwidth of the equivalent BPF is also 12 kHz. The Q-factor is increased by a factor of 6667, compared to an 80-MHz BPF centers at 8 GHz. After the second frequency-conversion, a BPF with 80-MHz bandwidth is employed to block the image frequency.

## Phase conjugate resonator based on degenerate OEPO

In the degenerate oscillation, we can see that the signal frequency is free from the cavity delay and only determined by the pump. This oscillation is also called half-harmonic generation. Note that the signal has continuous phase evolution in time domain, but in the spatial dimension, the phase has a hopping due to the frequency conversion process between the cavity *z=0* and *z=L*. This parametric process is actual a phase conjugate operator that reverses the signal phase from $-\varphi_{s}$ to $\varphi_{s}$. Based on this phase conjugate operation, we can implement a RF phase conjugate resonator, as shown in Fig. S3. The phase conjugate operator is based on second-order nonlinearity and the pump reverses the phase of the input signal. Though the frequency of the signal at *z=0* is unconditionally fixed at half of the pump, its phase is still sensitive to the cavity delay. Fortunately, we can find somewhere in the cavity in which the phase of the signal can be free from the cavity delay and varies synchronously with the pump signal. The site is located at *z=L/2*. Regardless of intensity change, we can obtain

$s\left(z=\frac{L}{2},t\right)=s\left(z=0,t+\frac{\tau}{2}\right)=\mathrm{Re}\left\{e^{-i\left(\omega_{s}t+N\pi\right)}\right\}$. (S1)

This equation suggests that the signal at *L/2* is unconditionally synchronized to the pump signal and its phase is free from the cavity delay even if the cavity delay may fluctuate due to the ambience variation and mechanical vibration.


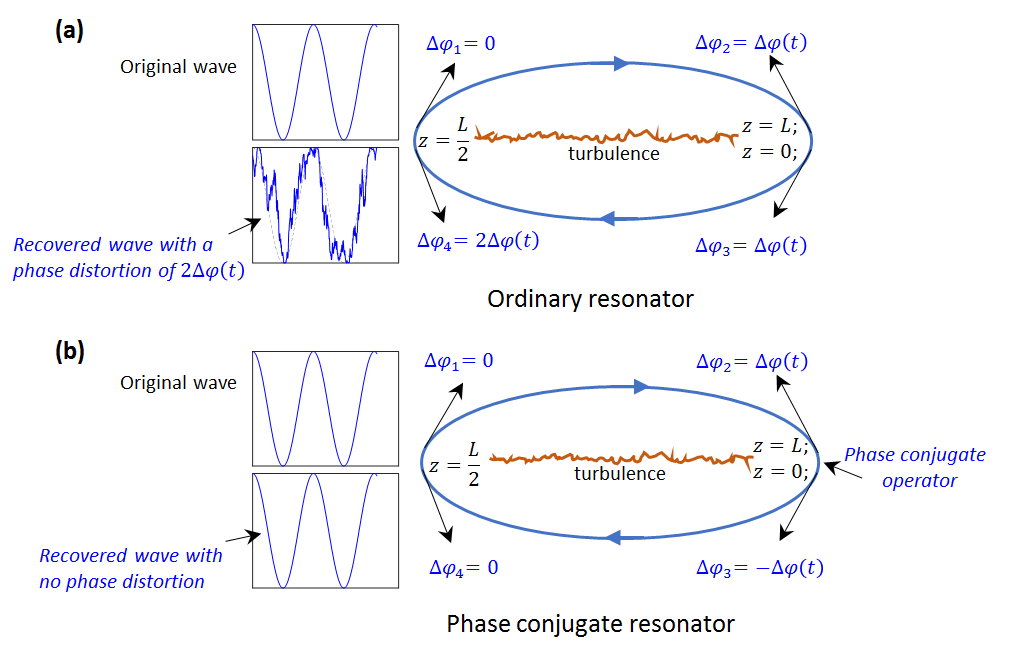


**Fig. S3.** A comparison between the conventional resonator and the phase conjugate resonator. $\varphi_{i}$ is the accumulated phase error at different position of the cavity, where i=1, 2, 3, 4. In conventional resonator, cavity turbulence would disturb the signal and leads to an unstable phase. In phase conjugate resonator, the sign of the phase error is reversed by the phase conjugate operator, so the phase error would be compensated automatically and the phase is unconditionally stable at the position of $z=\frac{L}{2}$.

A demonstration is implemented as shown in Fig. S4. The fiber in our demonstration is 5 km. An equivalent narrow BPF centers at 2.42 GHz with 12-kHz bandwidth is used to suppress the potential multi-mode oscillation. The accuracy of the frequency synchronization is first frequency-down-converted to 10 MHz, and then filtered out and measured by a frequency counter. Results are shown in Fig. S5. The fractional frequency instability, in terms of overlapping Allan deviation, is 1.3×10^−13^/9×10^−16^ at 1/10^4^ s averaging time. In the free running, directly delivery link, the long-term Allan deviation will increase along with the average time for the reason that link delay is sensitive to the ambient temperature fluctuation. A typical temperature coefficient of the single-mode fiber is 34 ps/km/℃. Despite of the small deterioration compared to the electrical noise floor, the Allan deviation in our scheme shares the same slope. This indicates that the link turbulence is compensated perfectly. Moreover, the small deterioration comes from backward Rayleigh scattering as the optical signal propagates in two directions.


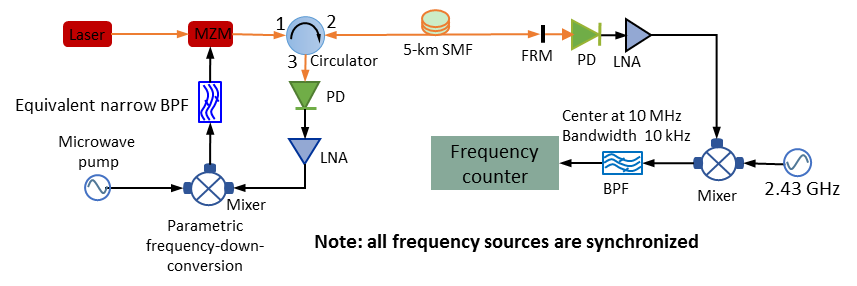


**Fig. S4.** Demonstration of phase-stable microwave transfer based on degenerate OEPO. In order to increase the measurement precision, the microwave is frequency-down-converted to 10 MHz by an external 2.43 GHz local oscillation. MZM, [Mach-Zehnder modulator](http://www.baidu.com/link?url=tGGWD9f3TWHxUMxPjdPtYoYp8dMxOJHEXxtGg8QGfxc4so7t8_Dt9eQ5JMZfkERGvGeN7RxXeaaZBbHQlWrXIGIbn7uqqyvlpHrBwLYxptu); SMF, single-mode fiber; FRM, [Faraday rotator mirror](http://www.baidu.com/link?url=9Ndtu4XtzBc32HQ7G-YY8HHAzB-dzOsDxcue2KdGYOe_Zrg1jnYyJTDDonQUKoU3GkBJuMbtFxTMvog6dRBo9_); PD, photodetector; LNA, low noise amplifier.


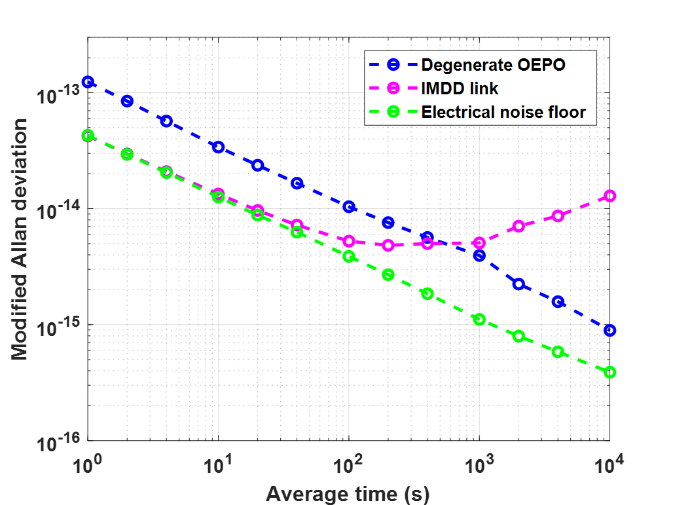


**Fig. S5.** The modified Allan deviation based on degenerate OEPO, and comparisons between the directly delivery scheme and electrical noise floor are implemented. IMDD: Intensity modulation direct detection.
